# (5*S*)-5-Methyl-3-phenyl-2-sulfanyl­idene-1,3-thia­zolidin-4-one

**DOI:** 10.1107/S1600536811051312

**Published:** 2011-12-07

**Authors:** Jun-Rong Jiang, Feng Xu, Zhong-Lu Ke, Li Li

**Affiliations:** a Department of Biological & Chemical Engineering, Taizhou Vocational & Technical College, Taizhou 318000, People’s Republic of China

## Abstract

In the title mol­ecule, C_10_H_9_NOS_2_, the 2-sulfanyl­idene­thia­zolidin-4-one mean plane and phenyl ring form a dihedral angle of 81.7 (1)°. In the crystal, C—H⋯π inter­actions link mol­ecules into helical chains in [010].

## Related literature

For related structures, see: Gattow *et al.* (1983 ▶); Rang *et al.* (1997 ▶). For applications of 2-sulfanyl­idene­thia­zolidin-4-one derivatives, see: Zidar *et al.* (2010 ▶); Powers *et al.* (2006 ▶).
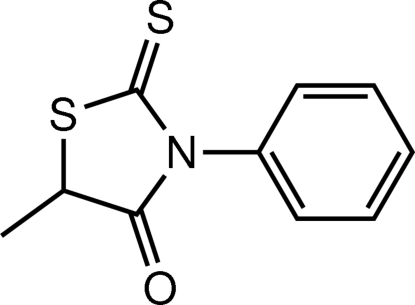


## Experimental

### 

#### Crystal data

C_10_H_9_NOS_2_
*M* *_r_* = 223.30
Orthorhombic, 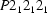

*a* = 6.8527 (4) Å
*b* = 8.6643 (5) Å
*c* = 17.5572 (15) Å
*V* = 1042.44 (12) Å^3^
*Z* = 4
Mo *K*α radiation
μ = 0.48 mm^−1^
*T* = 153 K
0.30 × 0.20 × 0.18 mm

#### Data collection

Rigaku AFC10/Saturn724+ diffractometer
Absorption correction: multi-scan (*CrystalClear*; Rigaku/MSC, 2008 ▶) *T*_min_ = 0.872, *T*_max_ = 0.919
9028 measured reflections
2777 independent reflections
2561 reflections with *I* > 2σ(*I*)
*R*_int_ = 0.029

#### Refinement

*R*[*F* ^2^ > 2σ(*F* ^2^)] = 0.028
*wR*(*F* ^2^) = 0.064
*S* = 1.00
2777 reflections
128 parameters
1 restraint
H-atom parameters constrained
Δρ_max_ = 0.30 e Å^−3^
Δρ_min_ = −0.17 e Å^−3^
Absolute structure: Flack (1983 ▶), 1155 Friedel pairs
Flack parameter: −0.01 (6)

### 

Data collection: *CrystalClear* (Rigaku/MSC, 2008 ▶); cell refinement: *CrystalClear*; data reduction: *CrystalClear* ; program(s) used to solve structure: *SHELXS97* (Sheldrick, 2008 ▶); program(s) used to refine structure: *SHELXL97* (Sheldrick, 2008 ▶); molecular graphics: *PLATON* (Spek, 2009 ▶); software used to prepare material for publication: *SHELXL97*.

## Supplementary Material

Crystal structure: contains datablock(s) global, I. DOI: 10.1107/S1600536811051312/cv5203sup1.cif

Structure factors: contains datablock(s) I. DOI: 10.1107/S1600536811051312/cv5203Isup2.hkl

Supplementary material file. DOI: 10.1107/S1600536811051312/cv5203Isup3.cml

Additional supplementary materials:  crystallographic information; 3D view; checkCIF report

## Figures and Tables

**Table 1:** Hydrogen-bond geometry (Å, °) *Cg* is the centroid of the C7–C12 ring.

*D*—H⋯*A*	*D*—H	H⋯*A*	*D*⋯*A*	*D*—H⋯*A*
C5—H5⋯*Cg*^i^	1.00	2.47	3.4321 (16)	162

Symmetry code: (i) 
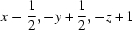
.

**Fig. 1.**
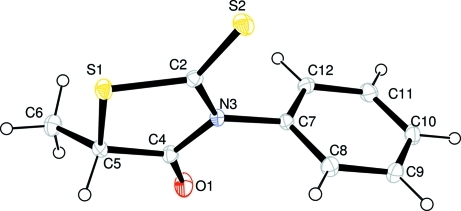
The molecular structure of (I), shown with 30% probability displacement ellipsoids.

## supplementary crystallographic information

### Comment

2-sulfanylidenethiazolidin-4-one derivatives are known as compounds with potential antifungal activities (Zidar *et al.*, 2010) and potential drugs-inhibitors of the HCV-RNA polymerase (Powers *et al.*, 2006). Herewith we present the title compound (I), which is a new 2-sulfanylidenethiazolidin-4-one derivative.

In (I) (Fig. 1), all bond lengths and angles are normal and correspond to those observed in the related compounds 3-(S)-(1-phenylethyl)-5-methyl-2-sulfanylidenethiazolidin-4-one (Rang *et al.*, 1997) and 5-methyl-2-sulfanylidenethiazolidin-4-one (Gattow *et al.*, 1983). The 2-sulfanylidenethiazolidin-4-one and phenyl rings form a dihedral angle of 81.7 (1)°. In the crystal structure, intermolecular C—H···π interactions (Table 1) link molecules into helical chains in [010].

### Experimental

To 54 ml of concentrated ammonia in an ice-salt bath was added 13.95 g(0.15 mol) of benzylamine. carbon bisulfide 19.5 ml(24.6 g,0.323 mol) was added dropwise over a period 2 h and stirring continued for 4 h.The dithiocarbamate precipitated was allowed to stand overnight. It was filtered(warning:filtered to be immediately used), washed with cold ether and dried by suction. The sodium 2-bromopropionate solution was prepared by 15.3 g(0.1 mol) of 2-bromopropionic acid in 9 ml of water and 3.5 g(0.0875 mol)of sodium hydroxide in 6 ml of water,and adding saturated NaHCO_3_ solution until the solution was basic.The sodium 2-bromopropionate solution was stirred, cooled to 273 K and the dithiocarbamate added by batch about 10 min.After the mixture was stirred for 1 h at the same condition,it was allowed to warm up to r.t. and stand 30 min.Then a hot solution of concentrated HCl plus water(40 ml+27 ml)was added to it.The mixture was boiled for 10 min and cooled to r.t.The precipitate was filtered, washed with cold water and little cold ethanol.The crude product was recrystallized from ethanol to yield 13.6 g(61%) yellow needle-like compounds.

### Refinement

H atoms were placed in calculated positions [C—H = 0.95-1.00 Å] and refined in riding mode, with *U*_iso_(H) = 1.2-1.5 *U*_eq_(C).

### Crystal data

**Table:**

C_10_H_9_NOS_2_	*F*(000) = 464
*M**_r_* = 223.30	*D*_x_ = 1.423 Mg m^−^^3^
Orthorhombic, *P*2_1_2_1_2_1_	Mo *K*α radiation, λ = 0.71073 Å
Hall symbol: P 2ac 2ab	Cell parameters from 3919 reflections
*a* = 6.8527 (4) Å	θ = 2.3–29.1°
*b* = 8.6643 (5) Å	µ = 0.48 mm^−^^1^
*c* = 17.5572 (15) Å	*T* = 153 K
*V* = 1042.44 (12) Å^3^	Block, colorless
*Z* = 4	0.30 × 0.20 × 0.18 mm

### Data collection

**Table:**

Rigaku AFC10/Saturn724+ diffractometer	2777 independent reflections
Radiation source: fine-focus sealed tube	2561 reflections with *I* > 2σ(*I*)
graphite	*R*_int_ = 0.029
Detector resolution: 28.5714 pixels mm^-1^	θ_max_ = 29.1°, θ_min_ = 2.6°
phi and ω scans	*h* = −9→9
Absorption correction: multi-scan (*CrystalClear*; Rigaku/MSC, 2008)	*k* = −11→10
*T*_min_ = 0.872, *T*_max_ = 0.919	*l* = −23→22
9028 measured reflections	

### Refinement

**Table:**

Refinement on *F*^2^	Secondary atom site location: difference Fourier map
Least-squares matrix: full	Hydrogen site location: inferred from neighbouring sites
*R*[*F*^2^ > 2σ(*F*^2^)] = 0.028	H-atom parameters constrained
*wR*(*F*^2^) = 0.064	*w* = 1/[σ^2^(*F*_o_^2^) + (0.0326*P*)^2^ + 0.086*P*] where *P* = (*F*_o_^2^ + 2*F*_c_^2^)/3
*S* = 1.00	(Δ/σ)_max_ = 0.014
2777 reflections	Δρ_max_ = 0.30 e Å^−^^3^
128 parameters	Δρ_min_ = −0.17 e Å^−^^3^
1 restraint	Absolute structure: Flack (1983), 1155 Friedel pairs
Primary atom site location: structure-invariant direct methods	Flack parameter: −0.01 (6)

### Special details

**Table:**

Geometry. All e.s.d.'s (except the e.s.d. in the dihedral angle between two l.s. planes) are estimated using the full covariance matrix. The cell e.s.d.'s are taken into account individually in the estimation of e.s.d.'s in distances, angles and torsion angles; correlations between e.s.d.'s in cell parameters are only used when they are defined by crystal symmetry. An approximate (isotropic) treatment of cell e.s.d.'s is used for estimating e.s.d.'s involving l.s. planes.
Refinement. Refinement of *F*^2^ against ALL reflections. The weighted *R*-factor *wR* and goodness of fit *S* are based on *F*^2^, conventional *R*-factors *R* are based on *F*, with *F* set to zero for negative *F*^2^. The threshold expression of *F*^2^ > σ(*F*^2^) is used only for calculating *R*-factors(gt) *etc*. and is not relevant to the choice of reflections for refinement. *R*-factors based on *F*^2^ are statistically about twice as large as those based on *F*, and *R*- factors based on ALL data will be even larger.

### Fractional atomic coordinates and isotropic or equivalent isotropic displacement parameters (Å^2^)

**Table:**

	*x*	*y*	*z*	*U*_iso_*/*U*_eq_	
S1	0.11471 (5)	0.55991 (4)	0.42516 (2)	0.02375 (9)	
S2	0.13997 (6)	0.70725 (4)	0.57733 (2)	0.02665 (10)	
O1	0.53389 (18)	0.27885 (14)	0.46834 (6)	0.0334 (3)	
N3	0.36082 (17)	0.47158 (13)	0.52694 (6)	0.0177 (2)	
C2	0.2145 (2)	0.57762 (16)	0.51556 (8)	0.0186 (3)	
C4	0.4056 (2)	0.37374 (17)	0.46629 (8)	0.0212 (3)	
C5	0.2751 (2)	0.40173 (16)	0.39840 (8)	0.0204 (3)	
H5	0.1937	0.3075	0.3899	0.025*	
C6	0.3913 (2)	0.4338 (2)	0.32607 (8)	0.0285 (3)	
H6A	0.4889	0.3525	0.3188	0.034*	
H6B	0.4569	0.5339	0.3306	0.034*	
H6C	0.3027	0.4357	0.2823	0.034*	
C7	0.4603 (2)	0.45370 (16)	0.59871 (7)	0.0184 (3)	
C8	0.3715 (2)	0.36714 (18)	0.65529 (8)	0.0246 (3)	
H8	0.2451	0.3249	0.6476	0.030*	
C9	0.4698 (3)	0.34305 (19)	0.72335 (9)	0.0285 (4)	
H9	0.4112	0.2829	0.7624	0.034*	
C10	0.6522 (2)	0.40624 (18)	0.73445 (8)	0.0280 (3)	
H10	0.7178	0.3909	0.7815	0.034*	
C11	0.7409 (2)	0.4923 (2)	0.67723 (9)	0.0274 (3)	
H11	0.8671	0.5348	0.6852	0.033*	
C12	0.6448 (2)	0.51620 (16)	0.60833 (8)	0.0226 (3)	
H12	0.7045	0.5742	0.5687	0.027*	

### Atomic displacement parameters (Å^2^)

**Table:**

	*U*^11^	*U*^22^	*U*^33^	*U*^12^	*U*^13^	*U*^23^
S1	0.02356 (17)	0.02662 (18)	0.02108 (17)	0.00623 (15)	−0.00460 (16)	−0.00175 (15)
S2	0.02865 (19)	0.02565 (18)	0.02565 (19)	0.00662 (16)	0.00106 (18)	−0.00678 (16)
O1	0.0377 (7)	0.0365 (7)	0.0259 (6)	0.0192 (6)	−0.0044 (5)	−0.0058 (5)
N3	0.0179 (5)	0.0197 (5)	0.0155 (5)	0.0002 (5)	−0.0008 (5)	−0.0004 (4)
C2	0.0180 (6)	0.0183 (6)	0.0194 (6)	−0.0016 (5)	0.0012 (5)	0.0008 (5)
C4	0.0225 (8)	0.0226 (7)	0.0186 (7)	0.0013 (6)	0.0004 (6)	−0.0003 (6)
C5	0.0230 (7)	0.0198 (7)	0.0186 (6)	0.0002 (6)	−0.0007 (6)	−0.0027 (6)
C6	0.0301 (8)	0.0347 (8)	0.0208 (7)	0.0031 (8)	0.0021 (6)	0.0006 (7)
C7	0.0216 (6)	0.0182 (7)	0.0154 (6)	0.0014 (5)	−0.0005 (5)	−0.0012 (5)
C8	0.0227 (7)	0.0286 (7)	0.0227 (7)	−0.0028 (7)	0.0021 (6)	−0.0003 (6)
C9	0.0374 (9)	0.0291 (8)	0.0189 (7)	−0.0009 (7)	0.0043 (7)	0.0031 (6)
C10	0.0355 (9)	0.0292 (8)	0.0193 (7)	0.0057 (7)	−0.0065 (7)	−0.0017 (6)
C11	0.0254 (7)	0.0281 (8)	0.0286 (8)	−0.0028 (6)	−0.0078 (6)	−0.0007 (6)
C12	0.0243 (7)	0.0205 (6)	0.0231 (7)	−0.0025 (6)	−0.0008 (6)	0.0023 (6)

### Geometric parameters (Å, °)

**Table:**

S1—C2	1.7350 (14)	C6—H6C	0.9800
S1—C5	1.8184 (15)	C7—C12	1.3856 (19)
S2—C2	1.6427 (14)	C7—C8	1.386 (2)
O1—C4	1.2043 (17)	C8—C9	1.387 (2)
N3—C2	1.3746 (17)	C8—H8	0.9500
N3—C4	1.3951 (18)	C9—C10	1.379 (2)
N3—C7	1.4412 (17)	C9—H9	0.9500
C4—C5	1.510 (2)	C10—C11	1.391 (2)
C5—C6	1.5245 (19)	C10—H10	0.9500
C5—H5	1.0000	C11—C12	1.393 (2)
C6—H6A	0.9800	C11—H11	0.9500
C6—H6B	0.9800	C12—H12	0.9500
			
C2—S1—C5	93.72 (7)	H6A—C6—H6C	109.5
C2—N3—C4	117.10 (11)	H6B—C6—H6C	109.5
C2—N3—C7	122.96 (11)	C12—C7—C8	121.67 (13)
C4—N3—C7	119.87 (12)	C12—C7—N3	119.76 (12)
N3—C2—S2	126.00 (10)	C8—C7—N3	118.49 (13)
N3—C2—S1	111.18 (10)	C7—C8—C9	119.05 (15)
S2—C2—S1	122.82 (9)	C7—C8—H8	120.5
O1—C4—N3	123.54 (13)	C9—C8—H8	120.5
O1—C4—C5	124.46 (13)	C10—C9—C8	120.14 (15)
N3—C4—C5	112.01 (12)	C10—C9—H9	119.9
C4—C5—C6	112.18 (12)	C8—C9—H9	119.9
C4—C5—S1	105.97 (10)	C9—C10—C11	120.47 (14)
C6—C5—S1	113.16 (10)	C9—C10—H10	119.8
C4—C5—H5	108.5	C11—C10—H10	119.8
C6—C5—H5	108.5	C10—C11—C12	120.01 (15)
S1—C5—H5	108.5	C10—C11—H11	120.0
C5—C6—H6A	109.5	C12—C11—H11	120.0
C5—C6—H6B	109.5	C7—C12—C11	118.64 (14)
H6A—C6—H6B	109.5	C7—C12—H12	120.7
C5—C6—H6C	109.5	C11—C12—H12	120.7
			
C4—N3—C2—S2	−178.34 (11)	C2—S1—C5—C4	−0.78 (10)
C7—N3—C2—S2	4.66 (19)	C2—S1—C5—C6	−124.12 (11)
C4—N3—C2—S1	1.17 (15)	C2—N3—C7—C12	−102.04 (16)
C7—N3—C2—S1	−175.83 (10)	C4—N3—C7—C12	81.04 (17)
C5—S1—C2—N3	−0.14 (11)	C2—N3—C7—C8	81.10 (18)
C5—S1—C2—S2	179.39 (9)	C4—N3—C7—C8	−95.83 (16)
C2—N3—C4—O1	177.98 (14)	C12—C7—C8—C9	0.2 (2)
C7—N3—C4—O1	−4.9 (2)	N3—C7—C8—C9	177.02 (13)
C2—N3—C4—C5	−1.81 (17)	C7—C8—C9—C10	0.8 (2)
C7—N3—C4—C5	175.29 (12)	C8—C9—C10—C11	−1.1 (2)
O1—C4—C5—C6	−54.3 (2)	C9—C10—C11—C12	0.5 (2)
N3—C4—C5—C6	125.48 (13)	C8—C7—C12—C11	−0.8 (2)
O1—C4—C5—S1	−178.26 (13)	N3—C7—C12—C11	−177.60 (13)
N3—C4—C5—S1	1.53 (14)	C10—C11—C12—C7	0.5 (2)

### Hydrogen-bond geometry (Å, °)

**Table:**

Cg is the centroid of the C7–C12 ring.

**Table:**

*D*—H···*A*	*D*—H	H···*A*	*D*···*A*	*D*—H···*A*
C5—H5···Cg^i^	1.00	2.47	3.4321 (16)	162

Symmetry codes: (i) *x*−1/2, −*y*+1/2, −*z*+1.
